# Effects of Dietary Fibres on Acute Indomethacin-Induced Intestinal Hyperpermeability in the Elderly: A Randomised Placebo Controlled Parallel Clinical Trial

**DOI:** 10.3390/nu12071954

**Published:** 2020-06-30

**Authors:** John-Peter Ganda Mall, Frida Fart, Julia A. Sabet, Carl Mårten Lindqvist, Ragnhild Nestestog, Finn Terje Hegge, Åsa V. Keita, Robert J. Brummer, Ida Schoultz

**Affiliations:** 1 School of Medical Sciences, Faculty of Medicine and Health, Örebro University, Södra Grev Rosengatan 32, 703 62 Örebro, Sweden; john-peter.ganda.mall@liu.se (J.-P.G.M.); frida.fart@oru.se (F.F.); julia.sabet@oru.se (J.A.S.); marten.lindqvist@oru.se (C.M.L.); robert.brummer@oru.se (R.J.B.); 2 Genetic Analysis AS, Kabelgata 8, 0580 Oslo, Norway; rn@genetic-analysis.com (R.N.); fth@genetic-analysis.com (F.T.H.); 3 Department of Biomedical and Clinical Sciences, Linköping University, 581 85 Linköping, Sweden; asa.keita@liu.se

**Keywords:** dietary fibres, prebiotics, intestinal permeability, NSAIDs, clinical trial, elderly, intestinal barrier function, gut health

## Abstract

The effect of dietary fibres on intestinal barrier function has not been well studied, especially in the elderly. We aimed to investigate the potential of the dietary fibres oat β-glucan and wheat arabinoxylan to strengthen the intestinal barrier function and counteract acute non-steroid anti-inflammatory drug (indomethacin)-induced hyperpermeability in the elderly. A general population of elderly subjects (≥65 years, *n* = 49) was randomised to a daily supplementation (12g/day) of oat β-glucan, arabinoxylan or placebo (maltodextrin) for six weeks. The primary outcome was change in acute indomethacin-induced intestinal permeability from baseline, assessed by an in vivo multi-sugar permeability test. Secondary outcomes were changes from baseline in: gut microbiota composition, systemic inflammatory status and self-reported health. Despite a majority of the study population (85%) showing a habitual fibre intake below the recommendation, no significant effects on acute indomethacin-induced intestinal hyperpermeability in vivo or gut microbiota composition were observed after six weeks intervention with either dietary fibre, compared to placebo.

## 1. Introduction

During the last decades lifespan has increased dramatically due to improved health and health-care. The global ageing phenomenon will have a major impact on health-care systems due to an increased prevalence of age-related diseases [1]. According to the National Board of Health and Welfare, gastrointestinal (GI) disorders are one of the most common side-effects that lead to hospitalizations of elderly persons in Sweden, often due to medications, such as non-steroid anti-inflammatory drugs (NSAIDs), commonly used for pain management [2]. The intestinal barrier has the function to absorb nutrients from the gut lumen while simultaneously acting as a barrier towards foreign substances, such as bacterial components and food derived toxicants. NSAIDs are known to induce an increased intestinal permeability across the epithelium [3]. Hence, therapeutic strategies able to increase the resistance of the intestinal barrier function against the negative effects of medication, such as NSAIDs, might have the potential to increase wellbeing. 

An adequate fibre intake has been emphasized as particularly important for the elderly, and national guidelines underline the necessity to increase the dietary fibre intake among the elderly population [4]. Prebiotics are dietary fibres that are known to have a positive effect on the gut microbiota by increasing the abundance of butyrate-producing bacteria and thus butyrate availability in the colon. Butyrate is the primary nutrient for colonic cells and has been suggested to play a role in maintaining and supporting intestinal barrier function [5,6,7]. Arabinoxylan, a dietary fibre originating from the wheat endosperm, has been shown to increase the amount of butyrate-producing bacteria in both animal and human studies [5,6,8,9]. Similarly, the dietary fibre oat β-glucan has been shown to increase beneficial bacteria in the gut and to enhance butyrate production in animal models [7,10]. However, the potential of dietary fibres to strengthen the intestinal barrier function in vivo in elderly individuals has, to our knowledge, not been extensively studied previously.

We performed a placebo-controlled parallel clinical trial in a general population of the elderly to investigate whether six weeks of oral supplementation of arabinoxylan or β-glucan could strengthen the intestinal barrier function and counteract a transient acute NSAID-induced hyperpermeability (e.g., indomethacin), using the in vivo multi-sugar permeability test. Furthermore, gut microbiota composition, inflammatory status and self-reported health was evaluated after dietary fibre intervention.

## 2. Materials and Methods 

### 2.1. Study Participants and Ethics

Community-dwelling individuals aged 65 years and older, representing the general population in the region of Örebro, Sweden, was recruited through advertisements in local and regional newspapers. Notification of interest was made through a web-based survey and information meetings were held at Örebro University. The study protocol was thoroughly presented and the research team was present for individual discussions and questions. Eligible participants were recruited after signing the consent form. A total of 60 eligible free-living elderly individuals were recruited (see inclusion and exclusion criteria in Table 1). The study received approval from the Regional Ethics Board in Uppsala (dnr 2015/357) and was conducted in accordance with the Declaration of Helsinki. The clinical trial has been registered in Clinicaltrials.gov (NCT03336385).

### 2.2. Trial Design

**Figure 1:**
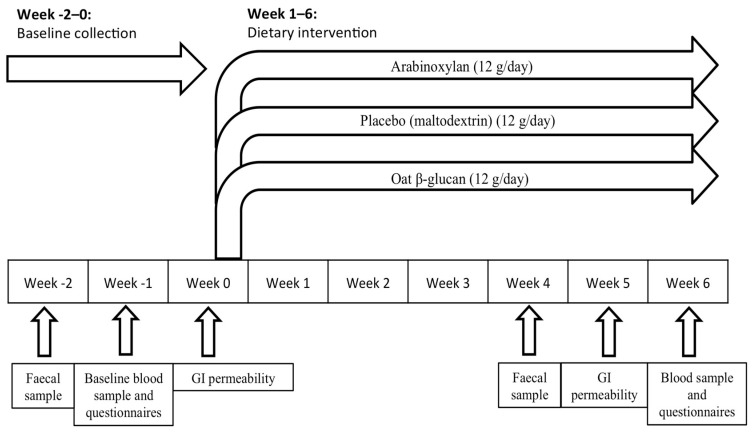
Schematic overview of the randomised clinical trial spanning a total of nine weeks, illustrating the time points for collection of faecal, blood, and urinary samples for gastro–intestinal (GI) permeability, in addition to questionnaire data before and after dietary fibre intervention.

The trial was designed as a randomised placebo controlled parallel clinical trial and was performed at Örebro University in Örebro, Sweden during October–December 2015. A schematic overview of the study design is outlined in Figure 1 and the participant flow is illustrated in the results section. The primary endpoint was change in intestinal barrier function as assessed by the multi-sugar permeability test. As there is limited knowledge regarding the size of the effect from in vivo prebiotic intervention on intestinal barrier function in the elderly, the sample size was based on previous successful studies using the multi-sugar permeability test and set to 20 participants per arm [11,12], giving a total number of 60 participants. The participants were instructed to maintain their normal dietary habits during the study period. Collection of baseline samples (urine, stool and blood) was performed prior to start of intervention at week −2, −1 and 0. After a daily supplementation at breakfast for six consecutive weeks, endpoint samples were collected during the two last weeks; more specifically urine and faecal samples were collected during week 5 (day 30–34) and blood and questionnaire data during week 6 (day 40–42). All samples were collected according to standardized operating procedures. 

All participants entered the intervention trial simultaneously. Participants were contacted two times per week during urine sample collection in order to ensure proper handling. In addition, the research team was available for questions through a dedicated study phone open 24/7 for urgent questions or for guidance through unforeseen events. The active study product consisted of Naxus^®^ (arabinoxylan, provided by Bioactor BV, Maastricht, The Netherlands) or Oatwell^®^ (oat β-glucan, provided by Swedish Oat Fibre, Väröbacka, Sweden) to a total weight of 12 g each. The placebo product consisted of maltodextrin with a similar appearance as the active study products, identically packaged and stored. The study products were packed in sachets and labelled by a third-party at Örebro University, Sweden, that had no other involvement in the clinical trial. Active and placebo sachets were packed identically along with the number of the subject and storing instructions. All products were blinded and block-randomised by a third-party not involved in conducting the clinical trial. For the research team, a blinded randomisation list was provided with the subject sequence and subject number. The research team allocated the study participants by randomly assigning them a study ID from the randomisation list of the pre-labelled study product. For each subject enrolled the date of enrolment and ID number of the trial subject was recorded. All other personnel and researchers involved in the study remained blinded to the study treatment until completion of the statistical analysis and discussion of the findings.

### 2.3. Data Collection

#### 2.3.1. Demographic Data

The following demographic information was collected: age, sex, BMI, and smoking habits, (see results section, Table 2). Habitual fibre intake was estimated through a food frequency questionnaire (FFQ) completed at study start. The FFQ has previously been validated in a Swedish population [13] and consists of 66 categories of food that evaluates the dietary pattern over the past year. 

#### 2.3.2. Medications

Information on prescribed medications taken during the six months preceding the study start was collected through a questionnaire. Current prescription of medications was documented in the case report form (CRF).

#### 2.3.3. Primary Intervention Parameters

**Figure 2:**
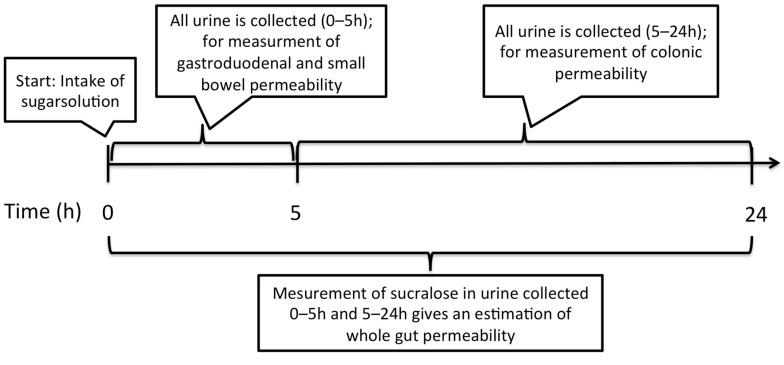
Illustration of the multi-sugar test. Urine is collected in two fractions, 0–5 h and 5–24 h, after intake of the sugar solution. By assessing sucrose in the 0–5 h fraction, gastroduodenal permeability is evaluated. Similarly, measurement of rhamnose and lactulose gives an estimation of small bowel permeability. Analysing sucralose and erythritol in the 5–24 h fraction will estimate the colonic permeability. Measurement of sucralose in both fractions of urine gives an estimation of the whole gut permeability. This study included a challenge to artificially increase intestinal permeability with the NSAID indomethacin.

GI permeability. A non-invasive previously validated multi-sugar test [3], quantifying 24 h urinary excretion of five different ingested sugars was used to assess gut permeability before (week -2) and after intervention with fibre supplements (week 5, days 32–34). The multi-sugar test estimates the permeability of four segments of the GI tract as outlined in Figure 2. GI permeability was assessed before and after challenge with the NSAID indomethacin (Gorch Fock Apotheke, Kiel, Germany) to investigate if dietary fibre supplementation could strengthen the intestinal barrier function and attenuate the effects of indomethacin. Blood samples were collected prior to study start for assessment of creatinine to ensure normal renal function. 

After an overnight fast, participants were instructed to drink a multi-sugar solution containing five sugars in the morning: 1 g Sucrose (Nordic Sugar, Örtofta, Sweden), 1 g Lactulose (Solactis, Jouy-en-Josas cedex, France), 0.5 g L-rhamnose (BioGaia, Stockholm, Sweden), 1 g Sucralose (Univar, Malmö, Sweden) and 1 g Erythritol (Ingredi, Stockholm, Sweden) dissolved in 150 mL of tap water. Urine was collected in two different fractions: fraction 1 containing the 0–5 h urinary output and fraction 2 the 5–24 h urinary output. The participants were not allowed to ingest any food or drinks except for water throughout the first urinary fraction (0–5 h) collection. During collection of urinary fraction 2 (5–24 h) the participants were allowed to drink and eat as normal, with the exception of caffeine-based products, alcohol, spicy food, drinks or sweets containing the same sugars as in the multi-sugar mix. Gastroduodenal and small intestinal permeability were reflected by the 0–5 h urinary sucrose excretion and the lactulose to L-rhamnose (L/R) ratio, respectively. In fraction 2 (5–24 h) the sucralose to erythritol (S/E) ratio represented colonic permeability. After collection of the 24 h urinary output, the total volume was quantified and urine aliquots were prepared by the participants using a built-in vacuum system in the collection jar (Sarstedt, Sweden) and stored at −20 °C. Before the second multi-sugar test, a 75 mg dose of indomethacin was ingested in the evening (9:30 pm), followed by an overnight fast and ingestion of a second dose of 50 mg at 5:30 am the following morning. The multi-sugar test was conducted 1h after the second dose of indomethacin. Samples were transferred to the lab within one week and stored at −20 °C until further analysis. Urinary sugars were analysed using liquid chromatography-mass spectrometry at Örebro University, Sweden. (See Appendix A for detailed description of the methodology.) Participants with a high creatinine value (women: >90 μmol/L, men: >105 μmol/L), indicating abnormal renal function, were excluded from further analysis (*n* = 1).

#### 2.3.4. Secondary Parameters

Assessment of gut microbiota composition. Material for collection of faecal samples was provided to each study participant one week prior to sampling and study start (week −1) and during week 5, days 30–34. The sample was collected at the participant’s home, instantly frozen at −20 °C and transported in a cooled condition to the laboratory within one week. For analysis of the microbial composition, total DNA was extracted from the faecal samples by using the QIAamp DNA stool mini kit according to the manufacturer’s instructions (Qiagen, Sollentuna, Sweden), coupled with an initial bead-beating step. Polymerase chain reaction (PCR) primers targeting the 16S rRNA gene were used to amplify 1180 base pair fragments containing the variable regions, V3–V9, as previously described by Casen et al. [14]. The PCR fragment was further used in a single-nucleotide extension reaction to label 48 bacterial probes in a panel (Table A1) and two internal controls. Together the 48 bacterial probes assess the presence of approximately 370 bacterial strains. Following hybridisation with barcoded magnetic beads (Luminex MagPlex beads, Luminex Inc., Austin, Texas, USA) the probe signals were detected on a Luminex MagPix. 

Questionnaire data. The Gastrointestinal Symptoms Rating Scale (GSRS) is a validated instrument, previously used to assess GI discomfort among the elderly [15,16]. The scale includes 15 questions divided into five symptom domains (e.g., reflux, abdominal pain, indigestion, diarrhoea and constipation). The symptoms are scored from 1 to 7 depending on their severity. Each domain was scored individually to assess the change in single symptoms after intervention while a total GSRS score (the mean score of all five symptom domains) was used to estimate the overall GI discomfort. Data regarding health-related quality of life was assessed through the EuroQol (EQ-5D) [17]. Psychological distress and the experience of stress were estimated through the validated Hospital Anxiety and Depression Scale (HADS) [18] and the Perceived Stress Scale (PSS) [19], respectively. (See Table A2 for more detailed information.) All questionnaires were completed before (during week −1) and at the end of intervention (week 6; day 40–42), as seen in the schematic overview of the randomised clinical trial (RCT) (Figure 1). All questionnaires were completed through a web-based survey with the exception of EQ-5D, which was completed using pen and paper. 

Blood biomarkers. After an overnight fast, the participants were instructed not to exercise in the morning, before collection of blood samples. Blood samples were collected before study start (week 0) and after intervention at week 6, day 40–42. Plasma was separated using standard operating procedures used in the daily routine at the Örebro University Hospital. C-reactive protein (CRP), oxidative stress (hydrogen peroxide) levels and cytokine expression were assessed using established methodologies; see Appendix A for detailed description.

### 2.4. Monitoring of Compliance and Adverse Events

All participants were asked to return any remaining study product after the intervention. Adherence was measured by calculating all remaining sachets. Participants who did not take the supplement on more than two non-consecutive days or consecutive days were considered non-compliant (*n* = 0). All adverse events following administration of study product and/or indomethacin intake were monitored until the end of the study. No serious adverse events (e.g., events resulting in death, immediately life-threatening events, medically significant events for any reason, events requiring or prolonging hospitalisation or resulting in persistent or significant disability/incapacity) were reported during or after the study.

### 2.5. Statistical Analysis

Baseline characteristics are displayed as mean (SD) for numerical variables and were compared using independent t-tests and Fisher’s exact test for categorical data. The median and interquartile range (IQR) was calculated for analysis of intestinal permeability (+/-indomethacin), inflammatory mediators, GSRS and HADS before and after intervention. An intention-to-treat (ITT) analysis was performed followed by a per-protocol analysis. Differences in intestinal permeability, gut microbiota composition (16S rRNA) and self-reported health after intervention with 12 g arabinoxylan, 12 g oat β-glucan or placebo were assessed using the non-parametric Mann-Whitney *U*-test for between-group analyses after subtraction of the baseline score. Gut microbiota composition before and after intervention was visualised by a Principal Coordinates Analysis (PCoA) based on Bray-Curtis distances between samples using packages vegan and labdsv in R. For assessment of differences within each intervention arm, Wilcoxon matched pair signed rank test was used. GI permeability data was not normally distributed as assessed by Shapiro-Wilk test and visualisation in histograms. To further explore intestinal permeability in relation to GI symptoms Spearman correlation analysis was performed. In sub-analyses, data were stratified based on a GSRS score > 2, depicting mild to severe GI symptoms. In addition a relief in GI symptoms (a reduction by 0.5 score points) was assessed after intervention, as well as changes in gut microbiota composition. In addition, sub-analyses were performed in order to identify whether participants experiencing a relief in GI symptoms after intervention also displayed an improved intestinal barrier function, as assessed by a reduction in indomethacin-induced intestinal hyperpermeability after intervention.

Gut microbiota composition was analysed in the sub-group focusing on changes in bacteria known from the literature for their butyrate producing capability, as well as bacteria with known probiotic effects (Table A1). Missing values (GSRS, *n* = 3; HADS, *n* = 3; PSS, *n* = 3; EQ-5D, *n* = 3) were replaced with the arithmetic mean of the completed items in the questionnaire, in accordance with the instructions of the questionnaire. All analyses were performed blinded. All data were stored in a common database and statistically analysed using R version 3.0 (New Zeeland) and GraphPad Prism 5 Software (GraphPad Software Inc., La Jolla, CA, USA). Degree of significance was indicated for datasets obtained as follows: *p*-value < 0.05 (*), *p*-value < 0.01 (**), *p*-value < 0.001 (***) or ns (non-significant).

## 3. Results

### 3.1. Participant Flow and Baseline Characteristics

**Figure 3:**
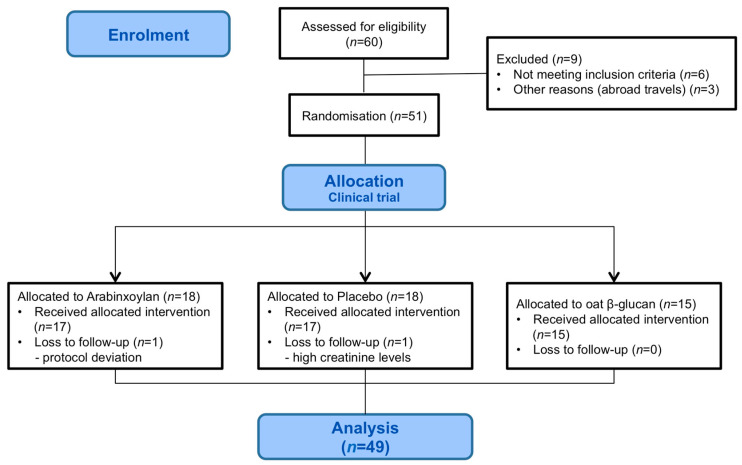
Flow chart showing the general participant flow throughout the study.

A total of 49 participants were included in the final analyses, corresponding to a treatment completion of 82% (17 arabinoxylan, 15 oat β-glucan, 17 placebo); dropouts and excluded participants can be viewed in Figure 3. The baseline characteristics did not differ significantly between the three intervention groups except for the medical category ‘others’, which contains several subcategories of diverse conditions. These types of conditions were present among 50% of the study participants in the placebo arm and among 15% in the two other arms (Table 2). The distribution of prescribed medications is shown in Table 2.

### 3.2. Primary Outcome

#### Assessment of Intestinal Permeability 

Initial analysis confirmed that intake of indomethacin significantly increased gastroduodenal permeability (*p* < 0.01), small intestinal permeability (*p* < 0.001) and colonic permeability (*p* < 0.01) in the whole study group. However, when analysing baseline separately according to the intervention group, indomethacin induced an increased colonic permeability only in the arabinoxylan treatment arm (*p* < 0.05) while small intestinal permeability was significantly increased (*p* < 0.05) in all intervention arms (Table 3). Gastroduodenal permeability was not significantly affected by indomethacin. Intervention with dietary fibres was found not to strengthen the intestinal barrier against indomethacin-induced hyperpermeability, as no significant differences were observed in the two treatment groups when compared to placebo (Table 3). GSRS score (Table 4) was not found to correlate with increased baseline (before indomethacin challenge) intestinal permeability and indomethacin-induced hyperpermeability (data not shown), neither before nor after intervention. In accordance with this observation, stratification of data based on a GSRS score of 2 did not reveal any changes in intestinal permeability after intervention when compared to placebo. 

### 3.3. Secondary Outcomes

#### 3.3.1. Assessment of Gut Microbiota Composition

**Figure 4:**
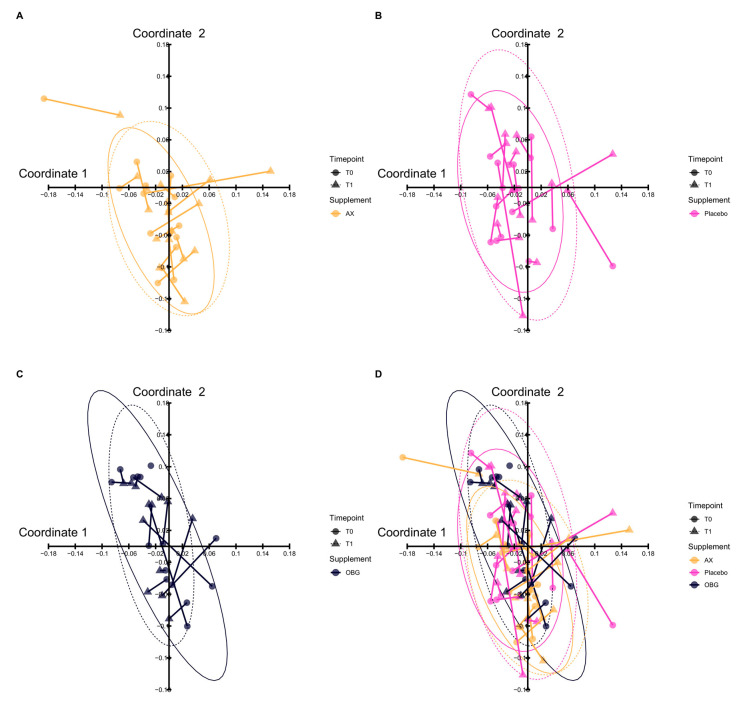
The composition of gut microbiota before and after intervention was visualized by Principal Coordinates Analysis (PCoA). The panels show gut microbial composition before and after intervention with (**A**) AX (Arabinoxylan) (**B**) Placebo and (**C**) OBG (Oat β-Glucan). In panel (**D)** an overlap of all interventions is displayed. The time points of sampling are indicated by shape: circle for T0, baseline and triangle for T1, end of study. To show the composition before and after intervention a line was drawn between samples from the same patient. One outlier per intervention group is not shown in the figure. As illustrated in panel (**D**), no separation of the groups could be observed after intervention.

By using the 48 labelling probes in the GA-map™ the gut microbiota composition was assessed. The initial ITT analysis showed no significant differences in the gut microbiota composition, i.e., beta diversity, before and after intervention, as visualised by a principal coordinates analysis in Figure 4, illustrating no separation of the three intervention groups after intervention. Neither did we observe any difference of Shannon indices (i.e., alpha diversity) at baseline and after intervention within the separate groups (data not shown). 

Moreover, we did not observe any significant changes when investigating participants experiencing a relief in GI symptoms. The generation of butyrate is dependent on the fermentation process of dietary fibres. Hence, as the intake of fibres was low in the study population we specifically assessed the abundance of 11 butyrate-producing bacteria (Table A1) before and after the dietary intervention. However, no significant changes could be observed, neither between the different dietary fibre intervention groups or when compared to placebo. 

#### 3.3.2. Evaluation of Gut Health, Wellbeing and Fibre Intake

No significant differences in GSRS, HADS, PSS or EQ-5D were identified before or after intervention when compared to placebo or within group analysis (Table 4). No differences were observed in any of the subdomains of the GSRS, illustrated in Table 4 by representable data from the two subdomains diarrhoea and constipation. Neither did stratification of data based on GSRS score ≥2 show any reduction in symptoms after intervention when compared to placebo. However, sub-analyses revealed a significant reduction in symptoms of diarrhoea (*n* = 6), from a GSRS score of 3.2, (IQR 2.23–3.75) to 1.5 (IQR 1.0–2.10) after intervention with arabinoxylan (*p* < 0.05), which failed to reach significance when compared to placebo. FFQ analysis revealed that 85% of those finishing the RCT had an insufficient fibre intake (g/day) at study start, accounting only for a median of 64.6% (IQR 50.6–83.8%) of the recommended intake (25.30 g/day) according to the Nordic Nutrition Recommendations (NNR). 

#### 3.3.3. Biomarkers of Inflammation and Oxidative Stress 

For all three groups the CRP levels were found to be within the normal range (<2 mg/L) and were not included in further analysis. Similarly, the levels of cytokines and oxidative stress were found to be close to the lower limit for detection (Table A3 and Table A4). Samples below detection limit were not included in the analysis.

## 4. Discussion

The present study focused primarily on the effects of dietary fibres on intestinal barrier function in elderly individuals, with the specific aim to investigate if daily supplementation with arabinoxylan or oat β-glucan could strengthen the intestinal barrier against acute NSAID-induced intestinal hyperpermeability compared to placebo. Before intervention, we observed that indomethacin increased small bowel permeability in all three-intervention arms, while increased colonic permeability was only observed in one of the arms and gastroduodenal permeability in none of the arms. This is in line with previous studies that have shown that indomethacin has a more profound effect on the small intestine [3].

The inability of the dietary fibres to attenuate indomethacin-induced hyperpermeability might be due to the fact that dietary fibres are mainly fermented into butyrate in the colon and might primarily have their effect there [5,6,7]. Although indomethacin significantly induced colonic hyperpermeability when pooling the pre-intervention colonic permeability data (*n* = 49), the main effect was seen on small intestinal permeability in the different intervention arms. This is in line with previous research [3,20] but the absence of an effect might be due to the low power in each arm. A cross-over design would have increased the power and reduced the inter-individual variation; however, these types of studies are, according to our experience, more difficult for elderly participants to continue and finish.

Neither did we observe any changes in baseline GI permeability before and after intervention as compared to placebo. This is in line with a recent study that showed no significant differences from baseline intestinal permeability after six weeks of arabinoxylan supplementation (7.5 g/day vs. 15 g/day) among lean and obese individuals [12]. Similarly, the starch pectin was found not to affect the intestinal barrier function in healthy adults and elderly individuals [21].

Furthermore, it is important to consider that only subtle treatment effects might be expected from prebiotics. The indomethacin dosage used in the present study was the standard dose for inducing small intestinal hyperpermeability in clinical studies [22,23,24]. However, indomethacin is currently not used as treatment in Sweden for any disease, but is widely used in several model systems for epithelial injury. 

The low intake of fibres among the elderly further makes the investigation of supplementation with dietary fibres important. However, according to our results supplementation with arabinoxylan or oat β-glucan do not alone have the possibility to affect baseline intestinal permeability, acute induced intestinal permeability or induce changes of the gut microbiota composition in elderly individuals. This is in contrast to previous studies that have shown that arabinoxylan and β-glucan supplementation can induce changes in the gut microbiota and increase the level of butyrate production, in human populations and animal models, respectively [9,10,25,26]. The absence of a significant effect on the gut microbiota after dietary supplementation in this study might be due to the fact that other fibres or a combination of fibres and probiotics might be more suitable for elderly individuals. Even though ageing is associated with a loss of *Faecalibacterium prausnitzii* [27] the non-observed effect might be due to geographical location. It has previously been shown that elderly and adult individuals in Sweden might have a higher abundance of the butyrate producing bacterium *Faecalibacterium prausnitzii* compared to other European countries [28]. Moreover, the gut microbiota composition in the present study was assessed by targeting the 16S rRNA region using 48 labelling probes. Thus, possible differences in the remaining gut microbiota after intervention, including levels of antibiotic resistant bacteria, were not investigated. To thoroughly understand the impact of arabinoxylan and oat-β-glucan on the gut microbiota composition, more advanced technology such as next-generation sequencing needs to be used.

The current study has some limitations. First of all, the collection of samples was not performed simultaneously on follow up out of concern for the elderly participants, and the collection was instead performed step wise; this could potentially have affected the outcome of the study although all samples were collected during a period of ten days for all study participants. Secondly, the study population was comprised of a heterogenous group, with an age range of 65-84 years, experiencing no GI symptoms, hence, potentially making it difficult to observe changes in gut microbiota and GI symptoms. It is therefore likely that the study design would have gained from a larger sample size and/or a more homogenous population regarding age or distribution of GI symptoms. Future studies should validate these results in a larger cohort, where samples are obtained within a shorter identical period in a more homogenous study population of elderly individuals. Moreover, the challenge of indomethacin might be too strong in relation to the modest effects on the intestinal barrier one might expect from intervention with dietary fibres. Hence, future studies should investigate if intake of dietary fibres can counteract the negative effects of long-term intake of milder NSAIDs, such as ibuprofen, amongst the elderly.

## 5. Conclusions

Despite the negative outcome of the study, our results highlight the importance of investigating dietary fibre supplementation in the elderly. Future clinical studies are needed in order to identify specific fibres able to strengthen the intestinal barrier in the elderly and counteract negative side-effects that are often associated with not only intake of NSAIDs, but also poly-pharmacy in the elderly. To identify cost-effective strategies is particularly important given the growing elderly population.

## Figures and Tables

**Table 1:** Inclusion and exclusion criteria for participation in the clinical trial.

**Inclusion criteria**
Informed consent signed by the study participant
Age ≥ 65 years
Mentally and physically fit to complete questionnaires during the study period
**Exclusion criteria**
Any known gastrointestinal disease with strictures, malignancies and ischemia
Inflammatory bowel diseases
Participation in other clinical trials in the past three months
Intake of medications known to change the inflammatory status (i.e., proton pump inhibitors, anti-inflammatory medications (including non-steroid anti-inflammatory drugs (NSAIDs))

**Table 2:** Demographic data of the study cohort.

	Arabinoxylan	Placebo	Oat β–Glucan	*p*–Value
	*n* = 17	*n* = 17	*n* = 15	
Male, *n* (%)	9 (55%)	9 (56%)	9 (60%)	ns
Female, *n* (%)	8 (47%)	8 (44%)	6 (40%)	ns
Age, years (median [IQR])	69.0 (66.0–71.5)	70.5 (67.0–78.3)	69.0 (66.0–72.0)	ns
BMI(median [IQR])	24.8 (23.0–29.1)	24.7 (22.1–27.2)	26.2 (22.4–28.0)	ns
Smokers	0	0	0	-
Baseline CRP levels (IQR)	0.7 (0.4–2.7)	0.6 (0.4–1.1)	0.8 (0.2–1.9)	ns
**Comorbidities**	%	%	%	
Cardiovascular diseases	41.2%	50.0%	33.3%	ns
Gut symptoms	35.3%	33.3%	20.0%	ns
Psychological& neurodegenerative morbidities	0%	11.1%	13.3%	ns
Others (lung, kidney, joints, eyes)	17.6%	50.0%	13.3%	*p* = 0.05
**Medications**	%	%	%	
Cardiovascular	41.2%	50.0%	33.3%	ns
Anti-inflammation	5.9%	0%	0%	ns
Gut regulating	29.4%	22.2%	20.0%	ns
Antibiotics	17.6%	16.7%	13.3%	ns
CNS-regulating drugs	5.9%	16.7%	6.7%	ns
Others	29.4%	50.0%	26.7%	ns
Multipharmacy (5 or more drugs)	17.6%	11.1%	0%	ns
Other GI regulators (probiotics, fibres etc)	17.6%	16.7%	0%	ns

**Table 3:** Intestinal permeability analysis of multi-sugar probes (μg/mL and ratios) before and after arabinoxylan (AX), placebo, and oat β-glucan (OBG) intervention, respectively, shown as median (IQR). No significant differences were found between the groups when compared to placebo.

	Pre-Intervention			After Intervention		
Study Arm	Baseline	Indomethacin-Challenge	*p*–Value	Baseline	Indomethacin-Challenge	*p*–Value
AX (*n* = 17)						
Gastroduodenal permeability [sucrose μg/mL]	3.37 (1.35–6.08)	3.86 (1.88–6.78)	ns	3.09 (1.42–8.91)	6.95 (2.67–13.04)	*p* < 0.05
Small intestinal permeability [L/R ratio]	0.038 (0.027–0.048)	0.056 (0.034–0.099)	*p* < 0.05	0.031 (0.020–0.056)	0.055 (0.045–0.125)	*p* < 0.05
Colonic Permeability [S/E ratio]	0.018 (0.009–0.027)	0.033 (0.024–0.04)	*p* < 0.01	0.015 (0.011–0.022)	0.029 (0.020–0.040)	*p* < 0.01
**Placebo (*n* = 17)**						
Gastroduodenal permeability [sucrose μg/mL]	1.69 (1.04–2.96)	2.26 (1.67–8.10)	ns	1.96 (0.77–6.53)	6.73 (2.03–11.99)	*p* < 0.05
Small intestinal permeability [L/R ratio]	0.029 (0.021–0.05)	0.05 (0.025–0.074)	*p* < 0.01	0.034 (0.019–0.049)	0.064 (0.028–0.098)	*p* < 0.01
Colonic permeability [S/E ratio]	0.023 (0.013–0.04)	0.028 (0.020–0.048)	ns	0.022 (0.017–0.030)	0.029 (0.017–0.046)	ns
**OBG (*n* = 15)**						
Gastroduodenal permeability [sucrose μg/mL]	2.22 (1.21–3.86)	2.79 (1.79–12.69)	ns	3.28 (1.23–9.18)	4.80 (1.35–13.05)	ns
Small intestinal permeability [L/R ratio]	0.027 (0.021–0.035)	0.056 (0.039–0.077)	*p* < 0.001	0.029 (0.018–0.040)	0.057 (0.042–0.090)	*p* < 0.001
Colonic permeability [S/E ratio]	0.016 (0.010–0.034)	0.029 (0.022–0.039)	ns	0.016 (0.008–0.05)	0.029 (0.014–0.038)	ns

**Table 4:** Questionnaire scores regarding gut health, psychological distress and stress at baseline and after intervention with arabinoxylan (AX), placebo, and oat β-glucan (OBG). Data presented as median (IQR).

	AX (*n* = 17) *			Placebo (*n* = 17)			OBG (*n* = 15)		
	Baseline	End of Study	*p*- value	Baseline	End of Study	*p*- Value	Baseline	End of Study	*p*- Value
**GSRS** - **Diarrha**	1.5 (1.0–2.8)	1.3 (1.0–1.9)	0.336	1.3 (1.0–2.7)	1 (1.0–2.7)	0.516	1.3 (1.0–2.0)	1.3 (1.0–2.3)	0.45
**GSRS** - **Constipation**	1.3 (1.0–2.6)	1.2 (1.0–2.9)	0.242	1.3 (1.2–3.2)	1.3 (1.0–2.8)	0.824	1.3 (1.0–1.7)	1.0 (1.0–2.0)	>0.99
**HADS**	4 (2–5)	3 ( 1–5.5)	0.106	5.5 (3.3–9.5)	5.5 (2.3–8)	0.529	3 (2–5)	5 (1–7)	0.55
**PSS**	8 (4.3–12)	6.5 (4.3–8.8)	0.319	9.5 (6.3–14)	10 (3–14)	0.814	8 (4–12)	6 (3–9)	0.28
**EuroQol** **VAS**	85 (76–95)	85 (80–90)	0.621	83 (80–94)	90 (76–95)	0.762	90 (81–95)	90 (80–95)	0.36
**EuroQol** **Index**	0.86 (0.79–0.86)	0.86 (0.79–0.86)	0.875	0.86 (0.76–1)	0.80 (0.76–1)	0.594	0.93 (0.86–1)	1 (0.86–1)	0.83

GSRS – Gastrointestinal Symptoms Rating Scale; HADS – Hospital Anxiety and Depression Scale; PSS – Perceived Stress Scale; * 17 included in the group, though there are missing values for HADS (*n* = 1), EuroQol-VAS (*n* = 1) and EuroQol-index (*n* = 2), leaving categories with 16, 16 and 15 participants, respectively.

## Abbreviations

GI: Gastrointestinal symptoms, NSAIDs: Non-steroid anti-inflammatory drugs, FFQ: Food frequency questionnaire, PCR: Polymerase chain reaction, GSRS: Gastrointestinal symptoms rating scale, EQ-5D: EuroQol, HADS: Hospital anxiety and depression scale, PSS: Perceived stress scale, CRP: C-reactive protein, IQR: Interquartile range, SD: Standard deviation, ITT: Intention to treat, PCoA: Principal Coordinates Analysis, AX: arabinoxylan, OBG: oat β-glucan.

## Appendix A

### Appendix A.1. Measurement of Sugar Probes in Urine Samples

Sample preparation and analysis. 1 mL of urine was centrifuged at 21,000 RCF for 25 min at a temperature of 4 °C. The supernatant was collected and 50 µL was transferred to LC vials to which 5 µL of 13C labelled lactulose and sucrose internal standard (Sigma Aldrich, MO, USA) was added. The urine aliquots were diluted to a volume of 1 mL with a composition of 80:20 acetonitrile: water. Before analysis, all samples were vortexed for 10 seconds and centrifuged for 15 min at 8000 RCF. Analysis was performed on an Acquity UPLC coupled to a Quattro Premier XE UPLC–MS/MS system (Waters Corporation, Milford, USA) with an atmospheric electrospray interface operating in negative ion mode. Analytes were separated on an Acquity BEH Amide column (1.7 µm, 2.1 × 100 mm) (Waters Corporation, Milford, USA). Column temperature was 50 °C, injection volume 10 µL, and flow rate 0.17 mL/min. An isocratic method was used with a mobile phase of 0.1% NH4OH in acetonitrile and water (70:30). Blanks and external standards were frequently injected during the analysis to control for carry over and monitor instrument stability. Mass-analysis was performed in the multiple reaction monitoring (MRM) mode by monitoring two product ions for each sugar analysed (341.16 > 100.80 and 341.16 > 160.80 for lactulose, 163.20 > 59.00 and 163.20 > 103.00 for rhamnose). Lactulose and sucrose were quantified using isotope dilution. Sucralose, erythriol, and rahmnose were quantified using 3-point standard addition curves, since no labelled standard was available for these analytes at the time of analysis. An in-house control sample was included in each batch.

### Appendix A.2. Measurement of Immune and Oxidative Markers in Blood

Systemic inflammation. C-reactive protein (CRP) is an acute-phase plasma protein and a marker of inflammation. CRP levels were assessed by the high-sensitivity immunoturbidimetric assay, CardioPhaseTM and analysed on the ADVIA 1800 chemistry system (SIEMENS Healthcare Diagnostics Inc., NY, USA). A normal CRP was defined as ≤2 mg/L according to clinical practice.

Oxidative stress. Oxidative stress was estimated by the validated FORT (Free Oxygen Radicals Test) colorimetric assay (Callegari, Parma, Italy), as previously described (5). Results are expressed as mmol/L H_2_O_2_.

Quantification of cytokines. Cytokines (IL-1β, IL-6, IL-8, IL-10, IL-2, IL-12p70, IFN-γ and TNF-α) were quantified using the Bioplex Pro™ Assay (BioRad, The Netherlands) according to manufacturer’s instructions. Samples were measured in the MagPix reader (BioRad) using MAGPIX xPONENT software (BioRad) and the concentration of the analyses was assessed by the Bio-Plex Manager version 5.0 (BioRad).

## Appendix B

**Table A1:** List of the bacterial targets of the 48 labelling probes in the GA-map^TM^.

Bacterial Probe	Phylum	Class	References *^,^#
Roseburia intestinalis/hominis *	Firmicutes	Clostridia	Flint HJ et al. 2002
Bacteroides vulgatus	Bacteroidetes	Bacteroidia	
Streptococcus agalactiae/Eubacterium rectale	Firmicutes	Bacilli/Clostridia	
Firmicutes/Tenericutes/Bacteroidetes species	Firmicutes/Tenericutes /Bacteroidetes species		
Firmicutes (Bacilli)	Firmicutes	Bacilli	
Enterococcus, Listeria	Firmicutes	Bacilli	
Butyrivibrio crossotus *	Firmicutes	Clostridia	Marius Vita, André Karch & Dietmar H. Pieper. 2017
Prevotella intermedia *	Bacterioidetes	Bacteroida	R. Krajmalnik-Brown et al. 2017
Firmicutes (Negativicutes)	Firmicutes	Negativicutes	
Bacteroides sp 4 3 47FAA/vulgatus	Bacteroidetes	Bacteroidia	
Veillonella, Helicobacter, Clostridia	Firmicutes	Negativicutes/Epsilonproteobacteria/Clostridia	
Enterobacter	Proteobacteria	Gamma Proteobacteria	
Ruminococcus champanellensis	Firmicutes	Clostridia	
Proteobacteria	Proteobacteria		
Bacteroides caccae	Bacteroidetes	Bacteroidia	
Clostridium leptum *	Firmicutes	Clostridia	Watada H. 2017
Ruminococcus bromii	Firmicutes	Clostridia	
Subdoligranulum variabile *	Firmicutes	Clostridia	Suzuki et al. 2014
Bacteroides pectinophilus	Bacteroidetes	Bacteroidia	
Coprococcus comes *	Firmicutes	Clostridia	De Vuyst et al. 2016
Parabacteroides johnsonii	Bacteroidetes	Bacteroidia	
Firmicutes (Clostridia) *	Firmicutes	Clostridia	Marius Vita, André Karch & Dietmar H. Pieper. 2017
Akkermansia municiphila ^#^	Verrucomicrobia	Verrucomicrobiae	Cani & de Vos. 2017
Bacterioides fragilis ^#^	Bacteroidetes	Bacteroidia	Zhi et al. 2018
Shigella/Echerichia	Proteobacteria	Gammaproteobacteria	
Prevotella nigrescens	Bacteroidetes	Bacteroidia	
Dorea longicatena	Firmicutes	Clostridia	
Bacteroides sp 2_2_4/ovatus/sp D1 ^#^	Bacteroidetes	Bacteroidia	Chen W et al. 2019
Eubacterium siraeum ^#^	Firmicutes	Clostridia	Putigani et al. 2017
Alistipes putredinis/onderdonki	Bacteroidetes	Bacteroidia	
Megasphaera micronuciformis/Dialister invisus	Firmicutes	Negativicutes	
Ruminococcus bicirculans	Firmicutes	Clostridia	
Eubacterium ventriosum *	Firmicutes	Clostridia	Kerckhof & Van de Wiele. 2012
Actinobacteria	Actinobacteria	Actinobacteria	
Enterobacter/Aeromonas	Proteobacteria	Gamma Proteobacteria	
Faecalibacterium *	Firmicutes	Clostridia	Flint et al. 2002
Providencia/Klebsiella/Pantoera	Proteobacteria	Gamma proteobacteria	
Lactobacillus ^#^	Firmicutes	Bacilli	Rastall et al. 2019
Collinsella aerofaciens	Actinobacteria	Actinobacteria	
Lachnospiraceae *	Firmicutes	Clostridia	Marius Vita, André Karch & Dietmar H. Pieper. 2017
Prevotella bivia/amnii	Bacteroidetes	Bacterioa	
Bacterioides acidofaciens	Bacterioidetes	Bacterioidetes	
Desulfitobacterium/Blautia	Firmicutes	Clostridia	
Roseburia inulinivorans *	Firmicutes	Clostridia	Marius Vita, André Karch & Dietmar H. Pieper. 2017
Streptococcus salivarius subsp.thermophilus ^#^	Firmicutes	Bacilli	Gibson et al. 2003
Bacteroides / Prevotella	Bacteroidetes	Bacteroidia	
Ruminococcus gnavus	Firmicutes	Clostridia	
Bacteroides dorei/sp. D4/sp. 9_1_42FAA	Bacteroidetes	Bacteroidia	

* Butyrate producing bacteria; ^#^ Probiotic bacteria.

**Table A2:** Overview of questionnaires.

Questionnaire	Variable	Scoring	Cut-off Values
**Gastrointestinal Symptoms Rating Scale (GSRS)**	Measure of gastrointestinal health, by five symptom domains: diarrhoea, indigestion, constipation, abdominal pain and reflux.	Fifteen questions rated on a 7-point scale using fixed respond alternatives.	0-2, no symptoms >2-3, mild symptoms >3-4, moderate symptoms >5, severe symptoms
**Hospital Anxiety and Depression Scale (HADS)**	Measure of psychological distress divided into two subscales: depression and anxiety.	Fourteen questions (7 questions per subscale) rated on a 4-point scale using fixed respond alternatives.	>7, signs of depression and/or anxiety
**Perceived Stress Scale (PSS)**	Measure of perceived stress, providing a total score.	Ten questions rated on a 5-point scale using fixed respond alternatives.	None available, the total score was used to estimate the total stress level
**EQ-5D-5L:** **EQ-Index**	Health-related quality of life. Mobility, self-care, usual activities, pain/discomfort and anxiety/depression Visual analogue scale (VAS)	5-point scale using fixed respond alternatives.	None available
**EQ-VAS**	recording subjective wellbeing	Respondents specify their subjective view on their wellbeing today on a scale from 0 (worst) to 100 (best).	

All questionnaires were completed online as a web survey with the exception of EQ-5D, which was completed using pen and paper.

**Table A3:** Expression of inflammatory markers analysed before and after intervention with arabinoxylan (AX), placebo, and oat β-glucan (OBG), displayed as median (pg/mL) and interquartile range (IQR).

		AX (*n* = 16)			Placebo (*n* = 17)			OBG (*n* = 15)	
	Baseline	End	*p*- Value	Baseline	End	*p*- Value	Baseline	End	*p*- Value
**IFN-y**	1.0 (0.0–4.7)	0.0 (0.0–7.8)	0.81	9.6 (0.0–44.3)	4.7 (0.0–39.6)	0.26	0.5 (0.0–13.0)	0.0 (0.0–17.7)	0.84
**IL-10**	4.3 (3.4–6.2)	4.5 (1.0–6.2)	0.75	4.4 (0.0–5.9)	5.0 (0.8–6.4)	0.25	3.9 (1.1–5.6)	4.9 (3.7–7.6)	0.32
**IL-12p70**	0.1 (0.0–0.5)	0.5 (0.0–1.2)	0.29	0.3 (0.0–0.5)	0.2 (0.0–0.5)	0.51	0.5 (0.1–0.8)	0.5 (0.0–0.5)	0.71
**IL-1b**	0.0	0.0	>0.99	0.0 (0.0–0.6)	0.0 (0.0–0.5)	0.26	0.0	0.0	0.75
**IL-2**	0.0	0.0	-	0.0	0.0	-	0.0	0.0	-
**IL-6**	1.3 (0.1–3.3)	1.4 (1.0–2.7)	0.98	2.1 (1.0–4.0)	1.4 (0.7–4.4)	0.94	1.4 (0.9–3.3)	1.5 (0.2–2.9)	0.76
**IL-8**	2.3 (1.4–3.7)	4.0 (1.7–6.8)	0.19	1.8 (1.3–3.6)	2.8 (1.7–4.0)	0.37	2.9 (1.6–5.9)	2.0 (1.5–4.0)	0.34
**TNF-a**	0.0 (0.0–1.9)	0.0 (0.0–2.8)	0.12	3.1 (0.0–10.9)	0.0 (0.0–13.0)	0.76	0.0 (0.0–1.9)	0.0	-
**CRP**	0.7 (0.4–2.7)	0.4 (0.2–2.3)	0.26	0.6 (0.4–1.1)	0.7 (0.3–1.2)	0.78	0.8 (0.2–1.9)	0.4 (0.2–2.4)	0.12

**Table A4:** The levels of reactive oxygen species (ROS), H_2_O_2_ mmol/L, were assessed in blood plasma collected before and after intervention with arabinoxylan (AX), placebo, and oat β-glucan (OBG). Data is shown with/without stratification below or above the Gastrointestinal Systems Rating Scale (GSRS) score 2. Results expressed as median and interquartile range (IQR).

Intervention Group	Baseline	End	*p*-Value
**AX (*n* = 13)**	2.01 (1.60–2.20)	1.7 (1.52–2.14)	0.106
GSRS diarrhoea /constipation score >2 (*n* = 5)	1.72 (1.52–2.20)	1.7 (1.52–1.96)	0.188
GSRS diarrhoea/constipation score <2 (*n* = 8)	2.07 (1.66–2.21)	1.77 (1.39–2.29)	0.383
**Placebo (*n* = 12)**	1.94 (1.84–2.19)	1.89 (1.71–2.03)	0.034 *
GSRS diarrhoea /constipation score >2 (*n* = 5)	2.18 (1.89–2.22)	2 (1.81–2.16)	0.437
GSRS diarrhoea /constipation score <2 (*n* = 7)	1.9 (1.8–2.0)	1.73 (1.54–2.0)	0.03 *
**OBG (*n* = 13)**	2.04 (1.87–2.15)	1.76 (1.57–1.99)	0.0005 ***
GSRS diarrhoea /constipation score >2 (*n* = 5)	2.0 (1.87–2.34)	1.8 (1.57–2.32)	0.125
GSRS diarrhoea /constipation score <2 (*n* = 8)	2.08 (1.67–2.17)	1.75 (1.41–1.93)	0.008 **

- H_2_O_2_ mmol/L <1.75 indicates no oxidative stress; - H_2_O_2_ mmol/L 1.75-2.35 indicates intermediate oxidative stress; - H_2_O_2_ mmol/L between 2.36-3.05 indicates oxidative stress; - H_2_O_2_ mmol/L 3.05< indicates strong oxidative stress; * = *p*-value < 0.05, ** = *p*-value < 0.01, *** = *p*-value < 0.001.
